# Potential role of doravirine for the treatment of HIV-1-infected persons with transmitted drug resistance

**DOI:** 10.1186/s12981-023-00503-5

**Published:** 2023-02-07

**Authors:** Soo-Yon Rhee, Jonathan M. Schapiro, Francesco Saladini, Maurizio Zazzi, Saye Khoo, Robert W. Shafer

**Affiliations:** 1 Department of Medicine, Stanford University, 1000 Welch Rd, Suite 202, Stanford, CA 94304 USA; 2 National Hemophilia Center, Sheba Medical Center, Ramat Gan, Israel; 3 Department of Medical Biotechnologies, University of Siena, Siena, Italy; 4 Molecular and Clinical Pharmacology, University of Liverpool, Liverpool, UK

**Keywords:** HIV-1, Antiviral therapy, Drug resistance, Mutations, Doravirine, Non-nucleoside RT inhibitor

## Abstract

**Background:**

Doravirine has a unique resistance profile but how this profile might increase its usefulness beyond first-line therapy in persons with susceptible viruses has not been well studied. We sought to determine scenarios in which doravirine would retain activity against isolates from ART-naïve persons with transmitted drug resistance (TDR) and to identify gaps in available doravirine susceptibility data.

**Methods:**

We analyzed published *in vitro* doravirine susceptibility data and applied the results to 42,535 RT sequences from ART-naïve persons published between 2017 and 2021. NNRTI drug resistance mutations (DRMs) were defined as those with a Stanford HIV Drug Resistance Database doravirine penalty score either alone or in combination with other mutations.

**Results:**

V106A, Y188L, F227C/L, M230L, and Y318F were associated with the greatest reductions in doravirine susceptibility. However, several NNRTI DRMs and DRM combinations lacking these canonical resistance mutations had > tenfold reduced susceptibility including G190E, one isolate with G190S, three isolates with L100I + K103N, one isolate with K103N + P225H, and isolates with L100I + K103N + V108I and K101E + Y181C + G190A. Of the 42,535 ART-naïve sequences, 3,374 (7.9%) contained a NNRTI DRM of which 2,788 (82.6%) contained 1 DRM (n = 33 distinct mutations), 426 (12.6%) contained 2 DRMs (79 distinct pairs of mutations), and 143 (4.2%) contained ≥ 3 DRMs (86 distinct mutation patterns). Among the 2,788 sequences with one DRM, 112 (4.0%) were associated with ≥ 3.0-fold reduced doravirine susceptibility while 2,625 (94.2%) were associated with < 3.0-fold reduced susceptibility. Data were not available for individual NNRTI DRMs in 51 sequences (1.8%). Among the 426 sequences with two NNRTI DRMs, 180 (42.3%) were associated with ≥ 3.0 fold reduced doravirine susceptibility while just 32 (7.5%) had < 3.0 fold reduced susceptibility. Data were not available for 214 (50.2%) sequences containing two NNRTI DRMs.

**Conclusions:**

First-line therapy containing doravirine plus two NRTIs is expected to be effective in treating most persons with TDR as more than 80% of TDR sequences had a single NNRTI DRM and as more than 90% with a single DRM were expected to be susceptible to doravirine. However, caution is required for the use of doravirine in persons with more than one NNRTI DRM even if none of the DRMs are canonical doravirine-resistance mutations.

**Supplementary Information:**

The online version contains supplementary material available at 10.1186/s12981-023-00503-5.

## Background

Doravirine (DOR) is an HIV-1 non-nucleoside RT inhibitor (NNRTI) approved in 2018 for the initial treatment of HIV-1 infection. It is also highly effective at maintaining virologic suppression with other antiretrovirals in persons without a prior history of virological failure (VF) [1–4]. DOR has a unique *in vitro* susceptibility profile, which has prompted its consideration for use in persons with some forms of NNRTI-associated transmitted drug resistance (TDR) [5, 6]. A clinical trial designed to assess DOR in persons with TDR caused by the three most common transmitted NNRTI drug-resistance mutations (DRMs), K103N, Y181C, and G190A, enrolled only nine persons [5].

Studies of amino acid mutations selected *in vitro* and in persons with VF while receiving DOR-containing antiretroviral therapy (ART) found that V106A, Y188L, F227C/L, M230L, and Y318F conferred the greatest reductions in DOR susceptibility. In 12 *in vitro* experiments V106A/M, V108I, H221Y, F227L/C/I, M230L, L234I, and Y318F were consistently reported to emerge [7, 8]. Among approximately 750 persons receiving DOR plus two NRTIs for initial ART, nine developed NNRTI DRMs associated with reduced DOR susceptibility, including eight isolates with > 90-fold reduced susceptibility [1, 2, 9]. The selected NNRTI DRMs included V106I (5 persons), F227C (5 persons), A98G (3 persons), V106A (2 persons), H221Y (2 persons), P225H (2 persons), Y318F (2 persons), V106M (1 person), E138G (1 person), and the 2-base pair mutation Y188L (1 person). Two of the nine persons developed just one NNRTI DRM (Y188L and Y318F). The remaining seven developed two or more NNRTI DRMs.

However, there has been no comprehensive analysis of *in vitro* DOR susceptibility data. To assess the potential usefulness of DOR for treating persons with TDR, we analyzed published *in vitro* DOR susceptibility data. We then examined a large set of sequences from ART-naïve persons published between 2017 and 2021 in the Stanford HIV Drug Resistance Database (HIVDB). Using the analysis of published DOR susceptibility data, we sought to determine scenarios in which DOR would retain activity against isolates from persons with TDR and to identify gaps in published DOR susceptibility data.

## Methods

### Published *in vitro* susceptibility data

Because *in vitro* susceptibility data were reported by four laboratories using different assays, we analyzed the results from each laboratory separately. The main analysis used data from the Merck Research Laboratory (MRL) which published more than twice the amount of data published by the other laboratories combined. For NNRTI DRM patterns with multiple available susceptibility results, we determined the median result. Results below  3.0-fold were reported as < 3.0-fold in accordance with the PhenoSense (Monogram BioSciences, South San Francisco) biological cut-off of 2.5-fold and the 3.0-fold cut-off used in a study that queried the Monogram database for isolates with single NNRTI DRMs [9, 10]. For the Monogram database single NNRTI DRM study, which reported the median susceptibility of multiple isolates, we treated the reported median as two results in our analysis. The studies were published between 03/2014 and 12/2020. Our analysis of these studies was completed by June 1, 2022.

### ART-naïve sequence dataset

We queried HIVDB for sequences of ART-naïve persons in studies published between 2017 and 2021. The ART histories of each of the persons was confirmed at the time each of the sequences were added to HIVDB. For each sequence we identified all NNRTI DRMs with a mutation penalty score for DOR according to version 9.0 (March 1, 2021) of the HIVDB drug-resistance interpretation program. This included 37 mutations with penalty scores when they occurred alone and/or in combination with other mutations including A98G, L100I/V, K101E/P, K103N, V106A/I/M, V108I, E138K, V179F, Y181C/I/V, Y188C/H/F/L, G190A/C/E/Q/S/T/V, H221Y, P225H, F227C/I/L/V, M230I/L, L234I, P236L, and Y318F. This list included mutations meeting one or more of the following criteria: (1) They were selected by DOR *in vitro* or in persons receiving DOR; (2) They were reported to reduce DOR susceptibility *in vitro*; (3) They appeared to contribute to reduced DOR susceptibility when they occurred in combination with another NNRTI-resistance mutation; and/or (4) They were suspected to possibly reduce DOR susceptibility because they occurred at a position at which one or more other mutations was associated with reduced DOR susceptibility.

To minimize the number of possible mutation patterns, we excluded 18 mutations that received a penalty score for one or more NNRTIs other than DOR including the highly polymorphic mutations K103R, E138A, and V179D and the following additional 15 mutations of which most are rare or have a minimal effect on NNRTI susceptibility: K101H, K103H/S/T, E138G/Q/R, V179E/L, Y181F/G/S, K238N/T, and N348I.

## Results

### Analysis of published *in vitro* susceptibility data

Eight studies reported 196 DOR *in vitro* susceptibility results [7–14]. Five studies reporting 136 results were published by authors at MRL [7, 9–12]. Three studies reporting 60 susceptibility results were published by other research groups—National Cancer Institute (NCI) in the U.S., University of Sienna (Italy) and McGill University (Canada) [8, 13, 14]. The MRL studies used the PhenoSense assay for 109 results and an MT-2 cell reporter gene assay for 27  results.

**Fig. 1:**
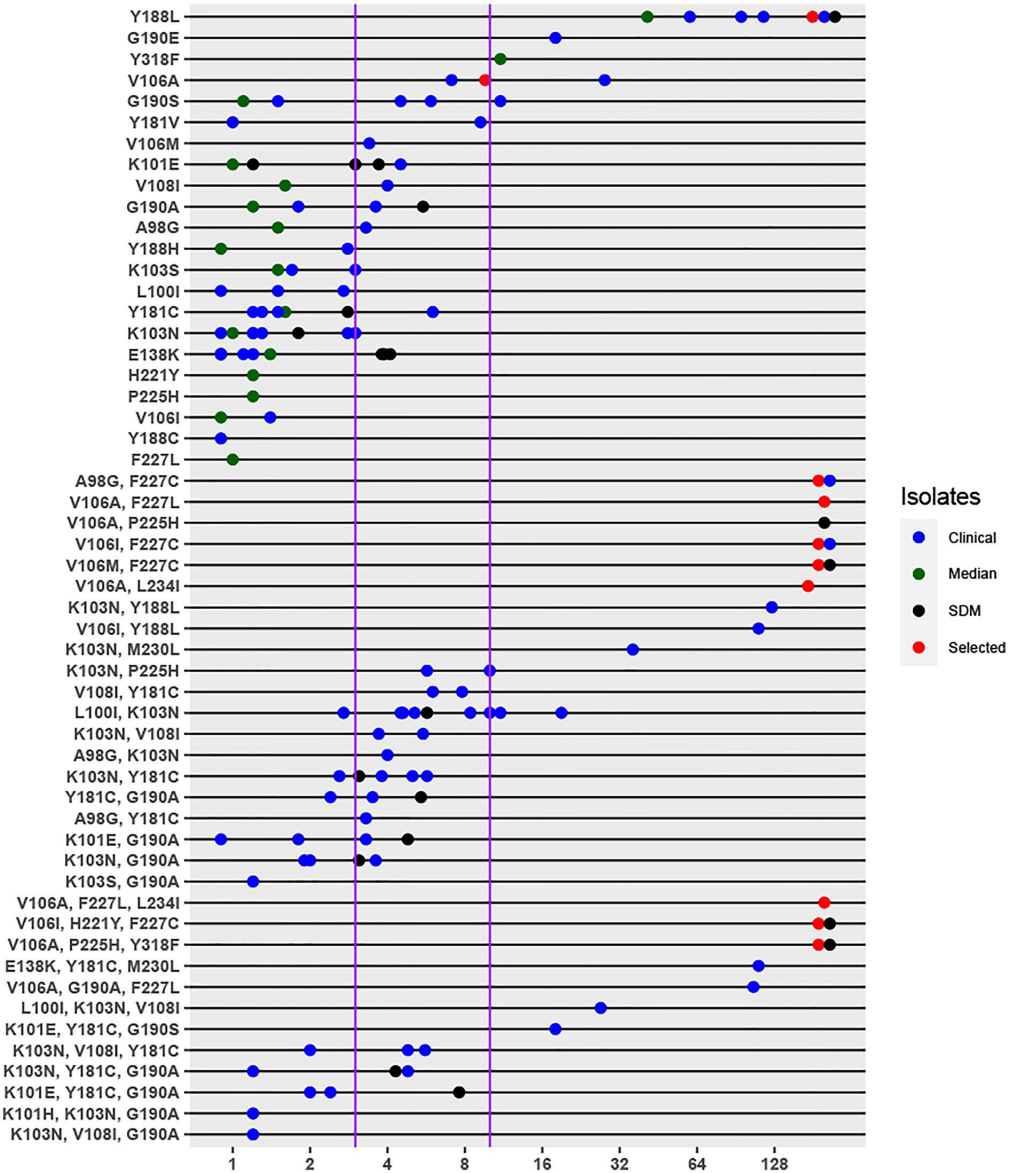
Doravirine in vitro susceptibility data for isolates with one, two, or three NNRTI-resistance mutations. The Y-axis indicates the pattern of mutations and the X-axis indicates the fold-reduction in susceptibility on a log_2_ scale. Isolates with a fold-reduced susceptibility < 1.0 were jittered about 1.0 whereas those with a fold reduced susceptibility > 128 were jittered at this level. Each of the results were published by the Merck Research Laboratory and included 109 results generated by the Monogram Biosciences PhenoSense assay and 27 results generated by an in-house MT-2 cell reporter gene assay. Blue circles indicate clinical isolates containing common patterns of NNRTI DRMs. Green circles indicate median fold reduced susceptibilities of viruses containing a single NNRTI DRM from a selection of viruses in the Monogram database [10]. Black circles indicate site-directed mutants containing common patterns of NNRTI DRMs. Red circles indicate site-directed mutants containing patterns of NNRTI DRMs that were selected either *in vitro* or in persons receiving doravirine. Vertical lines indicate 3.0-fold and  10.0-fold reduced susceptibility

Figure 1 shows the results published in the MRL studies according to type of isolate: (1) 89 were part of a Monogram Biosciences panel of clinical isolates containing common patterns of NNRTI DRMs (blue circles) [12]; (2) 21 were site-directed mutants containing common patterns of NNRTI DRMs including several selected in persons receiving DOR (black circles); (3) 16 results, each representing the median of viruses containing a single NNRTI DRM in the Monogram Biosciences database (green circles) [10]; and (4) 10 results on viruses containing patterns of NNRTI DRMs that were selected either *in vitro* or in persons receiving DOR (red circles).

Susceptibility data from the MRL studies were available for 86 isolates with 22 different single NNRTI DRMs, 46 isolates with 20 different pairs of NNRTI DRMs, and 20 isolates with 12 different patterns containing three NNRTI DRMs. Among the 22 different single NNRTI DRMs, four had been selected *in vitro* and/or *in vivo* by DOR and had a reduced susceptibility ≥ 3.0 fold including Y188L (> 64-fold; 8 results), Y318F (11-fold; 2 result), V106A (9.6-fold; 3 results) and V106M (3.4-fold; 1 result). Three other DRMs also had a reduction in susceptibility ≥ 3.0 fold including G190E (18-fold; 1 result), Y181V (5.1-fold; 2 results), and G190S (3.0-fold; 6 results).

Among the 20 different pairs of NNRTI DRMs, 9 patterns containing V106A, Y188L, F227C/L, or M230L had median reductions in susceptibility ≥ 36-fold. Eight patterns without any of these canonical DOR-associated mutations had median reductions in susceptibility ranging from 3.3 to 7.9-fold including K103N + P225H (7.9-fold; 2 results), V108I + Y181C (6.9-fold; 2 results), L100I + K103N (5.7-fold; 9 results), K103N + V108I (4.6-fold; 2 results), A98G + K103N (4.0-fold, 1 result), K103N + Y181C (3.8-fold, 5 results), Y181C + G190A (3.5-fold, 3 results), and A98G + Y181C (3.3-fold, 1 result).

Among the 12 patterns of mutations containing three NNRTI DRMs, the five containing V106A, F227C, or M230L had > 64-fold reductions in susceptibility. Two patterns lacking any canonical DOR-resistance mutations had > tenfold reductions in susceptibility including L100I + K103N + V108I and K101E + Y181C + G190S.

Additional file 1: Table S1 summarizes *in vitro* susceptibility data published by the McGill, Sienna, and NCI research groups. The McGill research group tested cultured viruses selected *in vitro* by DOR including V106A/I/M, V108I, H221Y, F227L, M230L, L234I, and Y318F in a 7 day multi-cycle assay with a read-out based on RT activity. Their fold reductions in susceptibility differed from those of the MRL group in that isolates with V108I + Y318F or H221Y + L234I were associated with high-level reductions in DOR susceptibility.

The Sienna group tested a panel of 10 site-directed mutants containing representative patterns of two to four NNRTI DRMs in a 48 h recombinant virus luciferase reporter gene assay. Although this panel was created prior to the approval of DOR, three isolates lacking canonical DOR-associated DRMs had > tenfold reductions in susceptibility including K103N + V179F + Y181C, V106I + Y181C + G190A + H221Y, and A98G + K101E + E138K + Y181C.

The NCI group tested 32 site-directed mutants associated with reduced NNRTI susceptibility in a 48-h recombinant virus luciferase reporter gene assay. Its results diverged from the other research groups in that K103N, E138K, and the uncommon NRTI-resistance mutation D67E had 7.0, 8.2, and a 70-fold reduction in DOR susceptibility, respectively.

### Predicted *in vitro* susceptibilities for ART-naïve sequences

HIVDB contained 42,535 one-per-person RT sequences from ART-naïve persons during the five- year period encompassing 2017 to 2021 reported in 168 published studies. Overall, 3374 (7.9%) had sequences with a mutation that had a penalty score for DOR (Additional file 2: Table S2). Of these, 2,788 (82.6%) contained a single NNRTI DRM (n = 33 different mutations), 426 (12.6%) contained two DRMs (n = 79 pairs of mutations), and 143 (4.2%) contained three or more DRMs (n = 86 patterns of mutations). Of the 3,374 sequences with a mutation that had a DOR penalty score, the most common subtypes were B (49.7%), C (12.3%), A (8.2%), CRF01_AE (8.0%), and CRF02_AG (7.1%). The distribution of sequences by region included Asia (31.5%), Europe (29.7%), Africa (26.1%), Latin America (11.9%) and North America (0.7%).

Among the 2,788 sequences with a single NNRTI DRM, 112 (4.0%) were associated with ≥ 3.0 fold-reduced DOR susceptibility, 2,625 (94.2%) were associated with < 3.0 fold-reduced susceptibility (Table 1). The 112 sequences associated with ≥ 3.0 fold-reduced susceptibility included three with the canonical resistance mutations V106A, Y188L, and Y318F and four with the non-canonical resistance mutations V106M, Y181V, and G190S/E. Susceptibility data were not available for 12 individual mutations including two canonical doravirine DRMs, F227C and M230L that occurred in five sequences and 10 additional mutations L100V, K101P, V179F, Y181I, G190Q, F227I/V, M230I, L234I and P236L that occurred in 46 sequences (Additional file 3: Table S3).

**Table 1:** Published Susceptibility for HIV-1 Isolates with a Single NNRTI-Associated Drug Resistance Mutation (DRM) Ordered by Frequency in the ART-Naïve Dataset^a^

NNRTI DRM^a^	Number of sequences	Fold-reduced susceptibility^c^ median _#tests_
V106I	1137	< 3.0_3_
K103N	817	< 3.0_9_
V108I	158	< 3.0_3_
G190A	123	< 3.0_5_
Y181C	102	< 3.0_7_
A98G	91	< 3.0_3_
K101E	76	< 3.0_6_
E138K	49	< 3.0_10_
H221Y	45	< 3.0_2_
Y188L^*^	40	106_8_
V106M	27	3.4_1_
G190E	17	18_1_
G190S^c^	11	3.0_6_
V106A^*^	8	9.6_3_
P225H	8	< 3.0_2_
F227L^*^	8	< 3.0_2_
Y188C	6	< 3.0_1_
Y318F^*^	5	11_2_
Y181V	4	5.1_2_
Y188H	3	< 3.0_3_
L100I	2	< 3.0_3_

^a^The table includes phenotypic susceptibility data published by the Merck Research Laboratory

^b^Susceptibility data were not available for 12 mutations including two canonical doravirine DRMs, F227C and M230L that occurred in five sequences and ten additional NNRTI DRMs, L100V, K101P, V179F, Y181I, G190Q, F227I/V, M230I, L234I and P236L, which occurred in 46 sequences. Mutations followed by an asterisk are canonical doravirine-resistance mutations

^c^The median fold-reduction in the Monogram single mutation database study was < 3.0 for G190S

Among the 426 sequences with two NNRTI DRMs, 180 (42.3%) were associated with ≥ 3.0 fold reduced DOR susceptibility, 32 (7.5%) were associated with < 3.0 fold reduced DOR susceptibility (Table 2). Susceptibility data were not available for 214 (50.2%) sequences including 57 containing one or more canonical resistance mutations or V106M, Y181V, or G190S/E. The remaining 157 sequences did not contain a DRM that individually was associated with ≥  3.0-fold reduced DOR susceptibility (Additional file 3: Table S3).

**Table 2:** Published Susceptibility for HIV-1 Isolates with Two NNRTI-Associated Drug-Resistance Mutations (DRMs) Ordered by Frequency in the ART-Naïve Dataset^a^

NNRTI DRM^b^	Number of sequences	Fold-reduced susceptibility median _#tests_
K103N, P225H	58	7.9_2_
L100I, K103N	32	5.7_9_
K101E, G190A	18	< 3.0_4_
K103N, Y181C	16	3.8_5_
K103N, V108I	15	4.6_2_
A98G, K103N	15	4_1_
K103N, G190A	14	< 3.0_4_
Y181C, G190A	10	3.5_3_
A98G, Y181C	9	3.3_1_
K103N, Y188L^*^	8	> 64_1_
V106I, Y188L^*^	5	> 64_1_
V108I, Y181C	5	6.9_2_
V106A, F227L^*^	4	> 64_1_
V106A, P225H^*^	2	> 64_1_
V106M, F227C^*^	1	> 64_2_

^a^The table includes phenotypic susceptibility data published by the Merck Research Laboratory

^b^Susceptibility data were not available for 214 sequences including 57 that contained ≥ 1 canonical DOR DRM or V106M, Y181V, or G190S/E. The remaining 157 sequences did not contain a DRM that was individually associated with ≥  3.0-fold reduced DOR susceptibility. Mutation patterns followed by an asterisk containing ≥ 1 canonical doravirine-resistance mutation

## Discussion

Several canonical DOR-resistance mutations alone or in combination with other mutations were associated with >  10.0-fold and often much greater reductions in DOR susceptibility including V106A, Y188L, F227C/L, M230L, and Y318F. Several other mutations were associated with greatly reduced susceptibility when they occurred in combination with canonical resistance mutations including A98G (with F227C), V106M/I (with F227C), P225H (with V106A), and L234I (with V106A). A98G, V106I, and P225H alone did not reduce DOR susceptibility. The only isolate with V106M alone with susceptibility data had 3.4-fold reduced susceptibility. L234I alone was not studied.

Although the isolates with the highest levels of reduced susceptibility in the MRL dataset usually had a canonical DOR resistance mutation, several other mutations and mutation combinations had reductions in susceptibility > 10.0-fold including G190E, one isolate with G190S, three isolates with L100I + K103N, two isolates with K103N + P225H, and one isolate each with L100I + K103N + V108I and K101E + Y181C + G190A. Several other combinations of two mutations were associated with median reductions in susceptibility of 3.0-fold to  8.0-fold.

The disparities in the DOR susceptibility results for viruses containing the same NNRTI-resistance mutations in the MRL dataset can result from several factors. First, two different assays were used. Second, some isolates were site-directed mutants whereas others were clinical isolates. Third, clinical isolates often have NRTI-resistance mutations which typically increase NNRTI susceptibility [15] and polymorphic mutations which can reduce or increase NNRTI susceptibility [16].

Our analysis indicates several gaps in existing susceptibility data for several individual mutations including V106M, Y181I/V, G190E, and L234I and suggests the need for additional data for viruses containing two or more non-canonical DOR-resistance mutations. Such data will provide a more complete picture of the potential usefulness of DOR for treating persons with TDR and for using DOR as an additional drug in persons with few other treatment options. Indeed, the amount of phenotypic susceptibility data for DOR is less than that of other NNRTIs. For example, as of July 2022, HIVDB contained 493 susceptibility results for rilpivirine from 17 publications and six scientific conferences even though rilpivirine has a much lower potential for use in persons with pre-existing NNRTI resistance.

DOR has been reported to display inhibitory quotients (trough concentration / antiviral IC_50_ in 100% human serum) of 68, 39, 27, and 25 against wildtype viruses and viruses with K103N, Y181C, and K103N + Y181C, respectively [11, 17]. This indicates that DOR may retain inhibitory activity against many viruses with low-level reductions in susceptibility such as that observed for most of the two-mutation NNRTI DRM patterns lacking canonical DOR-resistance mutations. However, the inhibitory activity of DOR against viruses from ART-naïve persons with TDR cannot necessarily be extrapolated to those persons with pre-treatment resistance who previously received an NNRTI because NNRTI-experienced persons are likely to have a more complex quasispecies containing more NNRTI DRMs than observed in ART-naïve persons.

## Conclusions

This study suggests that first-line therapy containing DOR plus two NRTIs is expected to be an effective regimen for treating most ART-naïve persons with TDR as more than 80% of TDR sequences had a single NNRTI DRM and as more than 90% with a single DRM are expected to be highly susceptible to DOR. However, among those with two NNRTI DRMs for which susceptibility tests were available, most had DRM patterns associated with a ≥ 3.0 fold-reduction in susceptibility. Therefore, our data suggest caution in the use of DOR for persons with more than one NNRTI DRM even if none of the DRMs are canonical DOR resistance mutations. This study also identifies several gaps in the available *in vitro* DOR susceptibility data that if filled would provide more confidence in the use of DOR beyond the approved indication for first-line therapy.

## Supplementary Information

**Additional file 1: **Additional file 1: **Table S1.** Word document. *In vitro* susceptibility data published by the McGill, Sienna, and NCI Research Groups.

**Additional file 2: **Additional file 2: **Table S2.** Excel file. Sequences from ART-naïve persons in the Stanford HIV Drug Resistance Database during the five-year period between 2017 and 2021 with a mutation that had a penalty score for DOR.

**Additional file 3: **Additional file 3: **Table S3.** Excel file. Patterns of DRMs present in sequences from ART-naïve in the Stanford HIV Drug Resistance Database during the five-year period between 2017 and 2021 annotated with phenotypic susceptibility data when available.

## Data Availability

The data supporting the findings of this study are available within the article and the Additional information files.
