# Supplementary material for: Potential role of doravirine for the treatment of HIV-1-infected persons with transmitted drug resistance
Source: AIDS Res Ther. 2023 Feb 7;20:8. doi: 10.1186/s12981-023-00503-5 (PMC9903540; doi:10.1186/s12981-023-00503-5)
Supplement: Supplementary file 1 — Additional file 1: Additional file 1: Table S1. Word document. In vitro susceptibility data published by the McGill, Sienna, and NCI Research Groups. [file 12981_2023_503_MOESM1_ESM.docx]

| **Additional file 1: Table S1. *In vitro* Susceptibility Data Published by the McGill, Sienna, and NCI Research Groups** | | |
| --- | --- | --- |
| AuthorYr | Drug-Resistance Mutations | Fold-Reduced Susceptibility  Median _#tests_ |
| Smith16  (NCI; Lab isolates) | L100I | <3.0_1_ |
|  | K101E | <3.0_3_ |
|  | K101P | <3.0_2_ |
|  | K103N | 7_1_ |
|  | V106A | 23_1_ |
|  | E138K | 4.5_3_ |
|  | Y181C | 3.1_1_ |
|  | Y181I | <3.0_1_ |
|  | Y188L | >64_1_ |
|  | G190A | <3.0_1_ |
|  | G190S | 6.6_1_ |
|  | H221Y | 7.2_1_ |
|  | M230L | >64_1_ |
|  | L234I | 10_1_ |
|  | L100I, K103N | 3_1_ |
|  | K103N, P225H | 38_1_ |
|  | K103N, Y181C | 18_1_ |
|  | V106A, F227L | >64_1_ |
|  | V106A, L234I | >64_1_ |
|  | V106A, F227L, L234I | >64_1_ |
|  | V106A, G190A, F227L | >64_1_ |
| Saladini21  (Sienna; recombinant molecular clones) | L100I, M230L | 6.2_1_ |
|  | K101E, Y181V | <3.0_1_ |
|  | K101E, G190S | 3.1_1_ |
|  | K101P, K103N | <3.0_1_ |
|  | L100I, K103N, H221Y | 4_1_ |
|  | K103N, V179F, Y181C | 22_1_ |
|  | H221Y, F227L, M230L | >64_1_ |
|  | A98G, K101E, Y181C, G190A | 32_1_ |
|  | A98G, K101E, E138K, Y181C | 14_1_ |
|  | V106I, Y181C, G190A, H221Y | >64_1_ |
| Brenner21  (McGill; Isolates resulting from in vitro passage) | V106A | 13_1_ |
|  | V108I | 8.4_1_ |
|  | L100I, E138K | 82_2_ |
|  | V106A, Y318F | >64_1_ |
|  | V108I, Y318F | >64_1_ |
|  | A98G, L100I, E138K | >64_1_ |
|  | V106A, V108I, Y318F | >64_1_ |
|  | V106A, F227L, Y318F | >64_1_ |
|  | V106M, V108I, Y318F | >64_1_ |
|  | V108I, E138K, M230L | 20_1_ |
|  | V108I, H221Y, L234I | >64_1_ |
|  | V106A, V108I, F227L, Y318F | >64_1_ |
|  | V106I, V108I, E138K, H221Y | >64_1_ |
|  | V108I, F227L, M230L, L234I | >64_1_ |
|  | V108I, F227L, M230L, Y318F | <3.0_1_ |
|  | V106A, V108I, F227L, M230L, Y318F | >64_2_ |
